# Effect of the Graded Silicon Content in SRN/SRO Multilayer Structures on the Si Nanocrystals and Si Nanopyramids Formation and Their Photoluminescence Response

**DOI:** 10.3390/ma14216582

**Published:** 2021-11-02

**Authors:** José Juan Avilés Bravo, Santiago Antonio Cabañas Tay, Liliana Palacios Huerta, Karla Esther González Flores, Javier Flores Méndez, Mario Moreno Moreno, Alfredo Morales Sánchez

**Affiliations:** 1 Electronics Department, INAOE, Apartado 51, Puebla 72000, Mexico; juan.aviles@inaoep.mx (J.J.A.B.); scabanastay@hotmail.com (S.A.C.T.); lilis.palacios@gmail.com (L.P.H.); mmoreno@inaoep.mx (M.M.M.); 2 Centro de Investigación en Materiales Avanzados S.C., Unidad Monterrey-PIIT, Apodaca 66600, Nuevo León, Mexico; karla.gonzalez@cimav.edu.mx; 3 Electronics Department, Benemérita Universidad Autónoma de Puebla, Puebla 72590, Mexico; xavier_snk@hotmail.com

**Keywords:** graded SRN/SRO multilayer, Si-nanocrystals, Si-nanopyramids, photoluminescence, LPCVD

## Abstract

Two multilayer (ML) structures, composed of five layers of silicon-rich oxide (SRO) with different Si contents and a sixth layer of silicon-rich nitride (SRN), were deposited by low pressure chemical vapor deposition. These SRN/SRO MLs were thermally annealed at 1100 °C for 180 min in ambient N_2_ to induce the formation of Si nanostructures. For the first ML structure (MLA), the excess Si in each SRO layer was about 10.7 ± 0.6, 9.1 ± 0.4, 8.0 ± 0.2, 9.1 ± 0.3 and 9.7 ± 0.4 at.%, respectively. For the second ML structure (MLB), the excess Si was about 8.3 ± 0.2, 10.8 ± 0.4, 13.6 ± 1.2, 9.8 ± 0.4 and 8.7 ± 0.1 at.%, respectively. Si nanopyramids (Si-NPs) were formed in the SRO/Si substrate interface when the SRO layer with the highest excess silicon (10.7 at.%) was deposited next to the MLA substrate. The height, base and density of the Si-NPs was about 2–8 nm, 8–26 nm and ~6 × 10^11^ cm^−2^, respectively. In addition, Si nanocrystals (Si-ncs) with a mean size of between 3.95 ± 0.20 nm and 2.86 ± 0.81 nm were observed for the subsequent SRO layers. Meanwhile, Si-NPs were not observed when the excess Si in the SRO film next to the Si-substrate decreased to 8.3 ± 0.2 at.% (MLB), indicating that there existed a specific amount of excess Si for their formation. Si-ncs with mean size of 2.87 ± 0.73 nm and 3.72 ± 1.03 nm were observed for MLB, depending on the amount of excess Si in the SRO film. An enhanced photoluminescence (PL) emission (eight-fold more) was observed in MLA as compared to MLB due to the presence of the Si-NPs. Therefore, the influence of graded silicon content in SRN/SRO multilayer structures on the formation of Si-NPs and Si-ncs, and their relation to the PL emission, was analyzed.

## 1. Introduction

Over the last several decades, silicon nanocrystals (Si-ncs) have been the subject of intensive study due to their potential applications as a strong light source [1,2,3,4]. Si-ncs embedded in dielectric matrices exhibit high luminescence and offer compatibility with Si-based technologies. Nevertheless, the development of this kind of device requires the control of the Si-ncs’ size to improve the charge injection [5,6]. Si-ncs are mostly obtained in single layers of Si-rich dielectric materials [7,8]. However, a broad Si-nc size distribution was obtained in these single layers. It is possible to control the size and density of Si-nc by employing a technique that allows the deposition of multilayer (ML) structures of Si-rich dielectric materials [9,10]. This ML approach makes it possible to engineer the bandgap energy of Si-ncs by optimizing the layer thickness and the composition (x, y or z < 2) of the SiO_x_/SiO_y_/SiO_z_ layers that form the ML structure. In fact, photovoltaic properties of Si-ncs have been obtained in a graded-bandgap ML structure where the size of the Si-ncs increases from the middle of the active region towards the electrodes [11]. This approach results in a decrease in the effective injection barrier at the electrodes and a concurrent increase in the charge carrier injection due to the presence of higher-efficiency small Si-ncs in the center of the active layer. Among the Si-rich dielectric materials, silicon-rich oxide (SRO) is one of the most studied. X-ray photoelectron spectroscopy studies (XPS) have shown that SRO is a multiphase material composed of a mixture of silicon dioxide (SiO_2_), off-stoichiometric silicon oxide (SiO_x_, x < 2) and elemental silicon, as stablished by the random bonding model [12,13]. It is well known that excess Si in the SRO layers agglomerates after a thermal annealing at high temperature, creating amorphous or crystalline Si nanoparticles (Si-nps) [14]. SRO layers are deposited by a large variety of techniques including: ion implantation of Si into SiO_2_ [15,16], magnetron sputtering of Si and SiO_2_ [17,18], laser ablation of Si targets [19], thermal evaporation of SiO [20,21], plasma-enhanced chemical vapor deposition (PECVD) [22,23] and low-pressure chemical vapor deposition (LPCVD) [24]. In LPCVD, silane (SiH_4_) and nitrous oxide (N_2_O) are used as reactive gases and the excess Si concentration is controlled by varying the ratio of the partial pressures produced by its fluxes, defined as R_O_ in Equation (1):

R_O_ = P(N_2_O)/P(SiH_4_) (1)

The excess Si content deposited into the SRO layers by LPCVD can be varied from 4 to 12.4 at.% for R_O_ values of 30 to 10, respectively [25]. Comparative studies focused on the photoluminescent (PL) properties of SRO layers deposited via different techniques have shown LPCVD as the technique that allows the strongest PL [26,27]. In addition, previous studies revealed that SRO-LPCVD layers with 5.5 at.% excess Si content, thermally annealed at 1100 °C for 180 min, emit the strongest PL [26]. The development of light sources based on SRO was shown to be possible through the use of metal-oxide-semiconductor (MOS) structures [28]. However, the electroluminescence (EL) response of such devices is usually inefficient due to the high electric field applied to obtain the carriers that tunnel through the oxide [29]. It has been shown that the presence of Si nanopyramids (Si-NPs) at the SiO_x_/Si-substrate interface improves the injection of charge carriers in indium tin oxide (ITO)/SiO_x_/Si-nanopyramid/p-Si/Al MOS devices emitting at lower voltages compared to those devices without the Si-NPs, as reported by Lin et al. [30]. The presence of interfacial Si-NPs produces specific zones of roughness at the SiO_x_/Si interface, which enhances the charge injection towards the Si-ncs through the Fowler–Nordheim (F-N) tunneling mechanism. They also make it possible to effectively extend the device lifetime by reducing the electric field away from the dielectric breakdown [31]. However, the voltages required to obtain the EL in those Si-NPs-based devices are still high, at about 65 V. The combination of Si-NPs and Si-ncs with gradual increases in the mean size can improve the charge injection to the luminescent centers through the use of an ML structure with SRO layers that have different Si concentrations.

Si-ncs and Si-NPs on the surface of Si-substrate can be obtained through the use of SRO layers with a specific amount of excess Si deposited by LPCVD and a subsequent thermal annealing [32]. Since the formation of the Si-NPs on Si substrates is very sensitive to the amount of excess Si in the SRO, there is a significant need to study the influence of Si concentrations on the size and density of Si-NPs and their PL responses. On other hand, silicon-rich nitride (SRN) is transparent to visible light and it has a band gap that is smaller than that of SiO_2_, facilitating the carrier injections needed for optoelectronic applications [33,34]. In MOS capacitors, Si nitride is used as buffer layer between the dielectric layer and the metal electrode to increase the efficiency and lifetime of the device [35]. Therefore, in this work, we studied the influence of graded silicon content in SRN/SRO multilayer structures on the formation of Si-NPs and Si-ncs and their relation to PL emission.

## 2. Materials and Methods

For this study, two different SRO ML structures were deposited on p-type silicon substrates ((100)-oriented) with resistivity values of 5–10 Ω-cm. The deposition was carried out by LPCVD using SiH_4_ and N_2_O as the reactive gases for the SRO films, and the partial pressure ratio (R_O_ = P_N2O_/P_SiH4_) was varied from 10 to 30 to change the excess Si in the ML structure. The ML structures consisted of 5 SRO layers capped by an SRN layer. The first ML structure (MLA) was fabricated by depositing layers of SRO_10_ (~10 nm), SRO_20_ (~20 nm), SRO_30_ (~20 nm), SRO_20_ (~20 nm) and SRO_10_ (~10 nm) over the Si substrate (R_O_ = 10/20/30/20/10/Si-substrate), where the sub-index in SRO indicates the R_O_ value. The second ML structure (MLB) was formed by SRO_30_ (~10 nm), SRO_20_ (~20 nm), SRO_10_ (~20 nm), SRO_20_ (~20 nm) and SRO_30_ (~10 nm) over the Si substrate (R_O_ = 30/20/10/20/30/Si-substrate). An SRN layer with R_N_ = 70 was deposited using SiH_4_ and NH_3_ (R_N_ = P_NH3_/P_SiH4_) as reactive gases. All layers were deposited at 750 °C. After deposition, the MLs were thermally annealed at 1100 °C for 180 min in ambient N_2_ to induce the Si agglomeration within the different SRO layers. The composition of the depth profile of thermally annealed MLs was analyzed by XPS using the Escalab 250Xi equipment (ThermoFisher, Waltham, MA, USA) produced by Thermo Scientific, with an Al Kα monochromatic source. The cross section high resolution transmission electron microscopy (HRTEM) and scanning TEM (STEM) images were obtained from ML structures using a JEOL JEM 2200 electronic microscope (Jeol Ltd., Tokyo, Japan). The PL spectra were obtained with a Horiba Jovin Yvon Fluoromax spectrometer (Horiba Ltd., Kioto, Japan) and were controlled by a computer. The samples were excited using a 300 nm (4.1 eV) wavelength and the PL measurements were scanned from 400 to 1000 nm with a resolution of 1 nm.

## 3. Results

### 3.1. Composition

**Figure 1:**
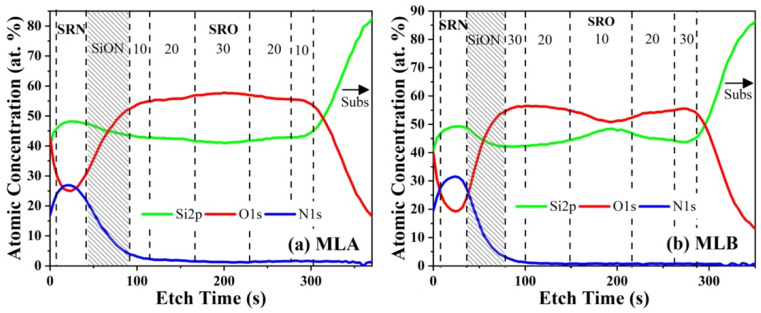
XPS depth profile for Si2p, O1s, N1s signal for (**a**) MLA and (**b**) MLB after thermal annealing.

The chemical compositions of the ML structures were studied using the XPS technique. The Si2p, O1s and N1s signals were obtained in XPS depth profiles by gradually etching the SRN/SRO ML structure to record the corresponding signals at different depths until the Si-substrate was reached. Figure 1a,b show the compositions of the depth-profiles of the MLA and MLB, respectively, where each SRO layer can be distinguished according to the Si content. For MLA, R_O_ = 10/20/30/20/10, the excess Si measured in each SRO layer was about 10.7 ± 0.6, 9.1 ± 0.4, 8.0 ± 0.2, 9.1 ± 0.3 and 9.7 ± 0.4 at.%, respectively. For MLB, R_O_ = 30/20/10/20/30, the excess Si was about 8.3 ± 0.2, 10.8 ± 0.4, 13.6 ± 1.2, 9.8 ± 0.4 and 8.7 ± 0.1 at.%, respectively. As observed in Figure 1a, for the MLA, the silicon content presented a gradual increase from the central layer (SRO_30_) towards the outer layers, while for MLB the opposite case occurred (Figure 1b). Abrupt changes in the Si and O profiles were not observed between SRO layers within the ML structure. This effect has previously been related to the high temperature of annealing (1100 °C), which promotes the diffusion of the Si and O atoms from the SRO layers with higher atomic percentage towards the SRO layers with lower percentages, generating the gradual interfaces. On the other hand, the Si content within the SRN layer for MLA and MLB was approximately 49.1 ± 0.2 and 50.0 ± 0.4 at.%, respectively. It was previously shown that, during the deposition of SRN, O atoms are incorporated into the single SRN films [36]. Moreover, the oxygen content varied with the R_N_ value; the higher the R_N_ value, the higher the O content. A diffusion of N, Si and O atoms towards the SRN/SRO interface was also observed due to the thermal annealing forming an oxynitride layer (SiON) with gradually increasing contents of the different elements. Similar results were reported in the literature for the formation of SiON at the interface of an SRN/SRO bilayer [37]. 

### 3.2. Structural Chracterization

**Figure 2:**
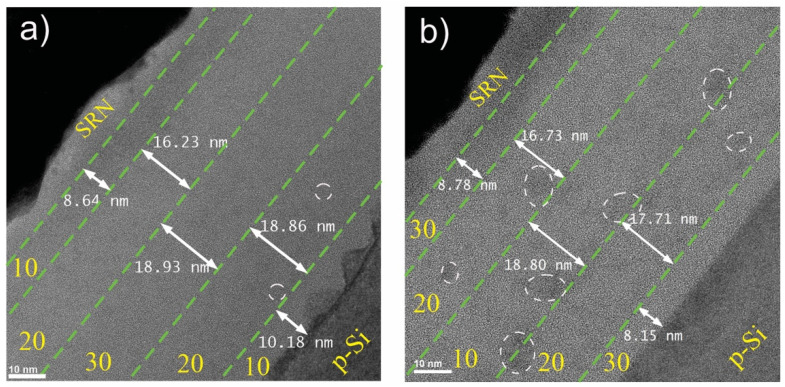
STEM cross-section micrograph of the (**a**) MLA and (**b**) MLB. The thickness and R_O_ value for each of the layers are indicated.

The microstructure of the MLs was studied with STEM and HRTEM to observe the presence of Si-ncs formation in the different ML structures. Figure 2a,b shows a micrograph of the cross-section of the MLA and MLB, respectively, where each of the SRO layers is labeled with its R_O_ value. The mean thickness of each SRO layer was obtained by means of statistical analysis of various STEM micrographs. The thickness of each SRO layer was less than expected. On the other hand, different dark zones (marked by circles) were observed in the micrographs, indicating the presence of Si-agglomerates within the ML structure, especially for MLB. The Si-NPs area could be clearly observed at the SRO_10_/Si-substrate (p-Si) interface of MLA (Figure 2a). Si-NPs were not present at the MLB, but Si-ncs were observed in the SRO layers with the highest excess Si (Figure 2b). 

**Figure 3:**
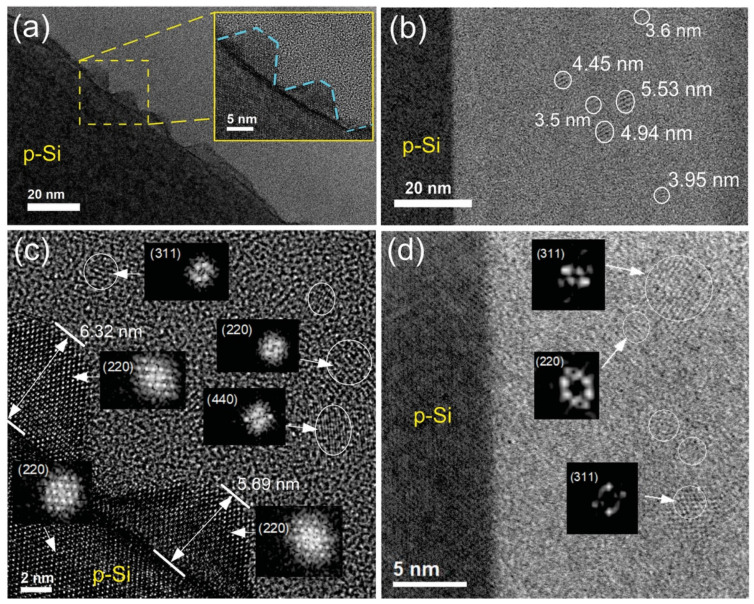
Cross-section HRTEM micrographs of the SRO/Si interface for (**a**) and (**c**) MLA and (**b**) and (**d**) MLB. Inset in (**a**) shows an enlarged image of the Si-nanopyramids.

To further analyze the formation of Si-ncs and Si-NPs, cross-section micrographs of the SRO/Si-substrate interface of the MLA and MLB were obtained by HRTEM, as shown in Figure 3a,b, respectively. For MLA, the formation of Si-NPs on the Si substrate can be observed. The inset of Figure 3a shows an enlarged image of the Si-NPs, which are marked with blue lines. For MLB, a homogeneous SRO/Si-substrate interface was observed, without the formation of Si-NPs, but with the presence of some Si-ncs in the vicinity, indicated with white circles in Figure 3b. Figure 3c,d show the SRO/Si-substrate interfaces of MLA and MLB, respectively, with higher resolutions (2 and 5 nm, respectively). An analysis of the diffraction patterns of Si-ncs and Si-NPs within the MLs is also included. The lattice spacing in the images was estimated using the Digital Micrograph software. Interplanar distances of 0.135 nm, 0.163 nm and 0.192 nm were measured, which corresponded to the (440), (311) and (220) crystalline planes of silicon, respectively. It should be noted that Si-NPs have the same crystalline orientation (220) as that of the Si substrate, as shown in Figure 3c. Further, the Si-NPs have base dimensions of between 8 and 26 nm, heights between 2 and 8 nm, and densities of ~6 × 10^11^ cm^−2^. The formation of Si-NPs in single SRO layers with the same excess Si but a longer thickness (580 nm) was previously reported [32]. The authors explained that the formation of Si-NPs could be described by a model of high-temperature diffusion and solid-phase crystallization. Since the SRO layers contain a large amount of excess Si, it diffuses to the surface of the Si substrate, where it agglomerates and crystallizes due to the thermal annealing. The Si-nucleation occurs more easily at the SRO/Si-substrate interface than in the bulk of the SRO due to the large strain at this interface. Then, the nucleation of the excess Si atoms reduce the strain and moves the whole system to a lower energy state. As observed in Figure 3, the formation of Si-NPs was very sensitive to the amount of excess Si in the SRO layer near the Si-substrate. According to the XPS results, in the MLA, the SRO layer next to the Si-substrate had an excess Si of 10.7 ± 0.6 at.%, while, in the MLB, the SRO layer had an excess Si of 8.3 ± 0.2 at.%. Therefore, it is possible that, during the deposition of the SRO_10_ at the SRO/Si-substrate interface, the Si atoms migrated along the Si-substrate surface quickly enough to orient themselves with the same crystal structure, thus allowing the Si-NPs to grow epitaxially.

**Figure 4:**
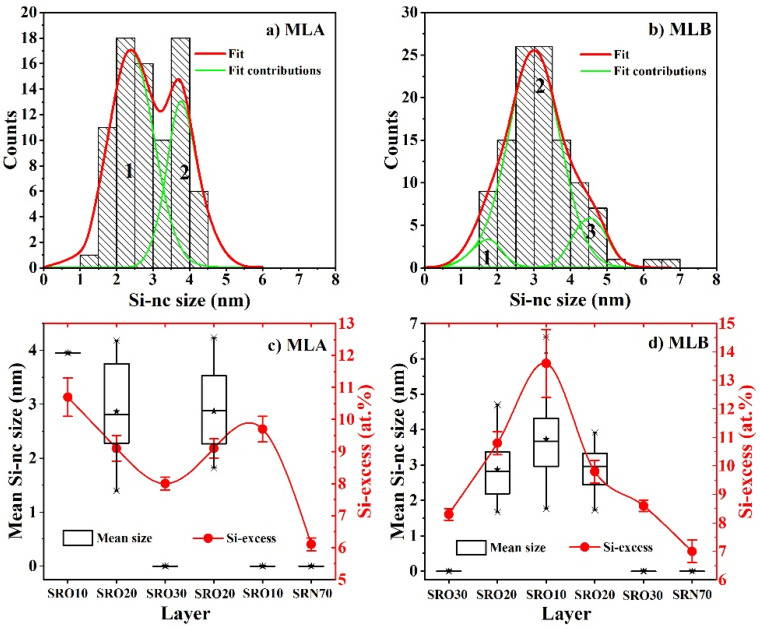
Histograms of Si-ncs sizes in (**a**) MLA and (**b**) MLB and the mean size of the Si-ncs vs. silicon excess of each SRO layer in (**c**) MLA and (**d**) MLB. Numbers in (**a**,**b**) indicates the Si-ncs size ranges.

By analyzing several HRTEM micrographs of the MLA and MLB, a statistical study of the size of the Si-ncs in the different SRO layers was performed. Figure 4 shows the histograms of Si-ncs’ sizes independently of the SRO layer. As we can see, there was a large distribution in the sizes of Si-ncs from 1 to 4.5 nm and 1.5 to 7 nm for the MLA and MLB, respectively. The presence of Si-ncs with larger sizes in the MLB could have been related to the high excess Si obtained in the central SRO_10_ layer, which was about 13.6 ± 1.2 at.%. For MLA, the sizes of Si-ncs could be separated into two ranges: (1) between 1 and 3.25 nm, and (2) between 3.25 and 4.5 nm (indicated by the green curves in Figure 4a). For MLB, they could be separated into three ranges: (1) between 1.5 and 2.25 nm, (2) between 2.25 and 3.75 nm, and (3) between 3.75 and 7 nm (indicated by the green curves in Figure 4b). Figure 4c,d show the mean sizes of the Si-ncs and the excess Si for each of the SRO layers. As we can see, an increase in the mean size of Si-ncs was observed as the excess Si within the SRO layer increased. Furthermore, the largest Si-ncs were formed in the middle layer of the MLB (see Figure 4d) where the highest silicon excess (13.7 ± 1.2 at.%) was present. On the other hand, in the MLA, Si-ncs were not formed in the SRO_10_ layer next to the SRN film, even though it contained a high concentration of excess Si (9.7 ± 0.4 at.%). This effect can be explained by the diffusion of N atoms and the formation of the SiON layer at the SRN/SRO interface. It was reported that N hinders the diffusion of Si atoms and prevents the phase separation in amorphous SiO_x_:N films [38]. Therefore, if the mobility of Si atoms is reduced, the formation of Si-ncs during thermal annealing is also reduced, resulting in small and non-crystalline Si-nanoparticles. The thickness, excess Si, mean size and density of Si-ncs obtained from XPS and HRTEM micrographs for each of the SRO layer are also summarized in Table 1.

### 3.3. Photoluminescent Properties

**Figure 5:**
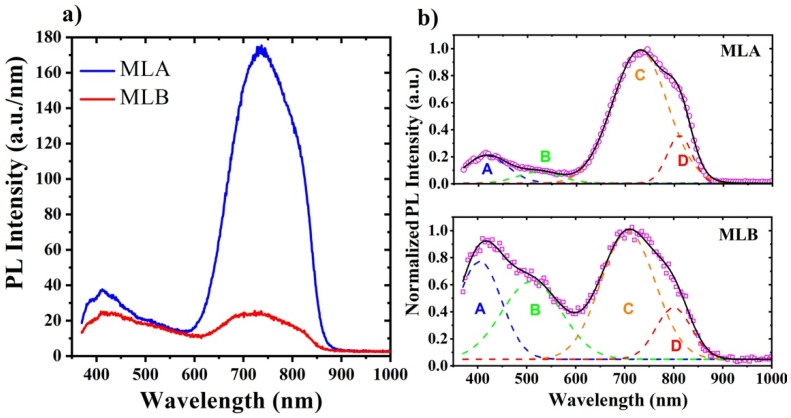
(**a**) PL spectra of MLA and MLB, and (**b**) deconvolution of the PL peaks for both MLs.

Figure 5a shows the PL spectra of the SRN/SRO MLs, where the PL intensity was normalized to the total thickness of each ML. 

It was observed that both MLs presented a broad emission spectrum, which could be divided into two main ranges: one from 370 to 590 nm and the other from 590 to 870 nm. The PL of SRO has been extensively studied for the development of Si-based light-emitting devices. Its origin was mainly related to luminescent centers such as O-based defects, band-to-band transitions in Si-ncs and radiative defects formed at the SRO/Si-ncs interface [4,19,29]. To observe the contributions of the different luminescent centers that were present in the MLs, the PL spectra were deconvoluted, as shown in Figure 5b. Four symmetrical bands centered around 415 (A band), 520 (B band), 730 (C band) and 810 nm (D band) were observed in both MLs. The violet-blue (400–460 nm) and green (520 nm) emission bands (A and B, respectively) were not related to the oxygen defects center (ODC) and the E’_δ_ (Si↑Si≡Si) defects, respectively [28,39]. In addition, it is possible that the SRN film contributed to the PL spectrum since it emits at 540 nm due to electronic transitions from the K^0^ centers to the =N − centers located near the valence band maximum (VBM), and at 420 nm due to electronic transitions from the K^0^ centers to the VBM [36,40]. However, the C (730 nm) and D (810 nm) bands were related to band-to-band transitions in Si-ncs due to the quantum confinement effects (QCE) [5], where their emission energy depended on their size. It was previously shown that the emission band can range from the near infrared to the blue by reducing the size of Si-ncs [41]. For the case of Si-ncs embedded in a SiO_2_ matrix, the energy varies according to the following expression [42]:

E_g_(d_ncs_) = E_g0_ + [5.83/(d_ncs_)^1.78^] (2)

where E_g0_ is the bulk silicon bandgap in eV, d_ncs_ is the Si-ncs diameter in nm and 5.83 is the confinement parameter. The emission energy, as calculated by eq. 2, for the largest Si-ncs of the MLA and MLB (3.5 to 4.5 nm), had values ranging from 1.73 eV (716 nm) to 1.51 eV (821 nm), which corresponded to the C and D bands. On the other hand, the difference in PL emission intensity between the two spectra was found to be quite significant. The MLA showed a stronger PL emission intensity, up to eight-fold, of the C and D emission bands as compared to MLB. The low intensity of PL emission in MLB may have been related to the presence of Si-ncs with sizes larger than 5 nm (see Figure 4d) and to the lower Si-ncs density than MLA. It was previously shown that Si-ncs with sizes larger than 5 nm do not contribute to PL, and also that a larger amount of Pb defects is generated at the interface of these Si-ncs, which are responsible for the quenching of PL emission [43]. On the other hand, the overall increase in the PL emission intensity of the MLA may have been due to the formation of the Si-NPs at the SRO_10_/Si-substrate interface. To explain this, it is necessary to mention that, in previous studies of LEDs [44,45], the release of the critical emission angle necessary to avoid total internal reflection (TIR), which facilitates a larger fraction of light emitted from the active region of an LED, has also been considered. Versatile methods of surface roughening have been introduced to improve the external quantum efficiency of LEDs. In our case, the roughness introduced by the Si-NPs at the SRO_10_/Si-substrate interface could avoid the TIR, which facilitated a larger fraction of light being emitted from the SRN/SRO structure to exit to the outside, thus improving the PL emission intensity. It is possible that this same effect will take place by applying an electrical excitation, and thus, it is possible that high electroluminescence will be obtained. Moreover, it is expected that the presence of Si-NPS with nearby Si-ncs will form preferential conduction paths and enhance the injection of charge carriers to the luminescent centers, and thus, reduce the turn-on voltage of the devices. Nevertheless, the electro-optical characterization of these SRN/SRO MLs will be performed in a future study. 

## 4. Conclusions

In this work, we reported the formation of Si-NPs and Si-ncs of different sizes in an SRN/SRO multilayer structure with a gradual silicon excess, as well as their relationship with PL. The formation of Si-NPs only occurred in the SRO/Si-substrate interface when an SRO layer with a high concentration of excess Si was next to the Si-substrate. Si-NPs with the same crystalline orientation as that of the Si-substrate were obtained. The mean size of Si-ncs in the multilayer varied according to the amount of excess Si in the SRO layers, where higher excess Si produced Si-ncs with a larger mean size. A wide distribution of Si-ncs’ sizes was obtained due to the conjunction of the SRO layers. Moreover, the PL emission of these structures was studied. An increase in PL emission (eight-fold more) was observed in the MLA due to the presence of Si-NPs at the SRO/Si-substrate interface as compared to MLB. In addition, a quenching in PL emission was observed for MLB due to the formation of Si-ncs with sizes larger than 5 nm in the middle layer of the SRO. In both MLs, a contribution of the SRN layer in the PL was observed with emission in the violet-blue bands.

## Figures and Tables

**Table 1:** Thickness, excess Si, mean size and density of Si-ncs obtained from XPS and HRTEM micrographs for each of the SRO layers.

Label	Layer Number	R_O_	R_N_	Excess Si (at.%)	Thickness (nm)	Si-ncs	
						Mean Size (nm)	Density (cm^−2^)
MLA	1	10	--	10.7 ± 0.6	10.16 ± 0.11	3.95 ± 0.20	6.79 × 10^11^
	2	20	--	9.1 ± 0.4	18.89 ± 1.25	2.86 ± 0.81	9.05 × 10^11^
	3	30	--	8.0 ± 0.2	19.96 ± 0.30	--	--
	4	20	--	9.1 ± 0.3	17.24 ± 1.55	2.87 ± 0.70	6.26 × 10^11^
	5	10	--	9.7 ± 0.4	9.67 ± 2.21	--	--
	6	--	70	6.1 ± 0.2	13.42 ± 3.33	--	--
MLB	1	30	--	8.3 ± 0.2	8.15 ± 0.74	--	--
	2	20	--	10.8 ± 0.4	17.72 ± 0.93	2.87 ± 0.73	1.31 × 10^11^
	3	10	--	13.6 ± 1.2	18.80 ± 0.85	3.72 ± 1.03	9.26 × 10^11^
	4	20	--	9.8 ± 0.4	16.79 ± 0.31	2.89 ± 0.61	5.85 × 10^11^
	5	30	--	8.7 ± 0.1	8.78 ± 0.56	--	--
	6	--	70	7.0 ± 0.4	11.51 ± 2.79	--	--

## Data Availability

The data supporting the reported results of this study can be made available from the corresponding author, upon request.
